# Olfactory dysfunction and altered cortical excitability in the mouse model of Fragile X Syndrome

**DOI:** 10.1186/s40659-024-00582-2

**Published:** 2025-04-24

**Authors:** Felipe Arancibia, Marcelo Rojas, Diego Becerra, Rocío Fuenzalida, Christian Cea-Del Rio, Jorge Mpodozis, Magdalena Sanhueza, Alexia Nunez-Parra

**Affiliations:** 1 Cellular Physiology Laboratory, Biology Department, Faculty of Science, Universidad de Chile, Santiago, Chile; 2 Neurophysiopathology Laboratory, Center for Biomedical and Applied Research, School of Medicine, Faculty of Medical Sciences, Universidad de Santiago de Chile, Santiago, Chile; 3 Neurobiology Laboratory, Biology Department, Faculty of Science, Universidad de Chile, Santiago, Chile

**Keywords:** Sensory processing, Autism spectrum disorder (ASD), *Fmr1* KO, Perceptual stability, Olfactory discrimination

## Abstract

Fragile X Syndrome (FXS) is the most common monogenetic cause of autism and inherited intellectual disability. A key feature of FXS symptomatology is altered sensory processing greatly affecting FXS individual’s life quality. Here, we use a combination of behavioral tests and slice physiology tools to study the neurophysiological alterations underlying aberrant sensory processing in the olfactory system of the FXS mouse model (*Fmr1* KO). We focused on the piriform cortex (PC), since it is in this brain region where olfactory information is integrated and ultimately decoded. Using a go-no go behavioral task we have found that *Fmr1* KO learn to discriminate between a rewarded and a not rewarded odorant but cannot distinguish complex odor mixtures, akin to what is found in the environment. Moreover, *Fmr1* KO long-term memory is impaired compared to control mice suggesting possibly cortical processing alterations. In addition, electrophysiological data from PC layer II neurons of *Fmr1* KO mice showed a hyperexcitable phenotype manifested by differences in active membrane properties and altered network connectivity. Taken together, our data suggest a possible causal link between the observed olfactory discrimination deficiencies in the *Fmr1* KO mouse and the altered physiology of PC.

## Introduction

Fragile X syndrome (FXS) is one of the most common causes of inherited intellectual disability and the most common monogenetic cause of autism spectrum disorder (ASD). FXS is produced by the repeat expansion of the CGG trinucleotide in the promoter region of the human *FMR1* gene located in chromosome X, which leads to hypermethylation and transcriptional silencing of the gene. Individuals with more than 200 CGG repetitions exhibit the full mutation and FXS [1, 2] and the absence of expression of the gene product fragile X messenger ribonucleoprotein (FMRP). FMRP is a selective RNA-binding protein exerting a critical regulation of pre- and postsynaptic proteins translation [3], as well as the synthesis and activity of various voltage-gated ion channels studied in animal models of the syndrome [4]. Due to the large number of targets that FMRP modulates, its absence generates a wide array of neurological symptoms, including atypical perception across sensory modalities (vision, audition, touch and olfaction) [5–8].

Despite the fact that individuals with FXS exhibit olfactory hyperreactivity [5, 7] the neuronal basis underpinning olfactory processing deficiencies have been poorly explored. The scarce scientific evidence obtained using genetically manipulated mouse, where the *Fmr1* gene was knocked out *(Fmr1* KO) [9], have revealed a higher odor detection threshold [10] and also olfactory discrimination deficits [11, 12], when compared to control mice. Interestingly, it appears that *Fmr1* KO animals can discriminate between sufficiently different olfactory cues but have trouble discriminating olfactory information when odor cues are similar [11]. How is the olfactory cortex of KO animals separating neuronal activity patterns to allow coding and discrimination of olfactory cues, is not known. Moreover, studies using complex odor mixtures composed by several odorants, that are more representative of their natural environment of mice, are also lacking.

Briefly, olfactory processing begins when volatile odorant molecules enter the nose and interact with receptors expressed in olfactory sensory neurons located in the nasal epithelium. These neurons project to the glomerular layer of the olfactory bulb, where they make their first synapse with mitral (MC) and tufted cells [13]. MC in turn project directly to the piriform cor­tex (PC) bypassing the thalamus [14]. PC principal neurons synapse onto each other forming an interconnected network encoding the identity of an olfactory stimulus by an ensemble of specific neurons that are co-activated in response to inputs from MC [15, 16]. Thus, the PC is suggested to store odor memories [15, 17] and as the region where the inhaled molecules features are translated into perceptual odor-objects [16]. In this context, there is vast evidence indicating that sensory cortices in the *Fmr1* KO mice model generate aberrant sensory stimulus representation [4, 6, 18–21] due to hyperexcitable neuronal networks state, suggesting that PC may undergo a similar alteration. Thus, here, we explored whether *Fmr1* KO mice exhibit a disequilibrium in perceptual stability and memory deficits, and whether the olfactory cortex exhibits physiological alterations that might play a role in the observed cognitive differences. Using a go-no go behavioral protocol, we found that although *Fmr1* KO successfully learns a binary discriminatory task, they cannot discriminate between complex odorant mixtures. Moreover, *Fmr1* KO also shows long-term memory impairment that is accompanied by hyperexcitation and altered active membrane properties of the principal neurons in the PC. Thus, our data suggests that deficiencies in cortical processing of odor objects due to abnormal excitability properties of the network could underpin the alterations in olfactory discrimination observed in the *Fmr1* KO mice.

## Methods

### Animals

C56BL/6 and *Fmr1* KO mice were obtained from The Jackson Laboratory and bred in the animal facility of the Biology Department, Faculty of Sciences, Universidad de Chile. Animal care and experimental procedures followed the regulations of the Institutional Committee on Animal Care and Use of the Universidad de Chile. For the behavioral experiments, 2–4 months old male mice were used. The mice were kept under a constant 12-hour light/dark cycle. Water and pelleted food were provided *ad libitum* before the experiments.

### Behavioral training

For the go-no go experiments mice were water-deprived for 48 h, but food was still provided *ad libitum*. The daily intake of water was regulated to maintain mice weight close to 85% of the weight recorded prior to deprivation. Subsequently, through a standard operant conditioning protocol [22], mice were trained daily in the olfactometer. First, they undergo a ‘Begin’ program [23], where the mice were rewarded with 5 µL of water when they inserted their snouts into the odor sampling port of the operant conditioning chamber and licked the water delivery tube. Later, the mice were rewarded for licking the tube for a progressively longer period (at least once per 0.5 s intervals, with the number of intervals increasing from 1 to 4) after an odor pulse of isoamyl acetate 1% v/v diluted in mineral oil was presented. The duration of the odor pulse also increased from 0.2 s to 1.5 s progressively through the blocks. The first part of the Begin program was 20 trials long, whereas the second part consisted in 8 blocks of 20 trials each. The mouse had to complete the 8th block of the Begin program for at least three consecutive days before moving into the go-no go tasks.

In these tasks animals were trained to distinguish between a rewarded (CS+) and an unrewarded (CS-) olfactory stimulus. The training session typically consisted in 10 blocks of 20 trials each (200 trials in total), and the CS- and CS + were presented an equal number of times in a randomized order within a block. The test starts when a water-deprived mouse places its snout in the odor port and the vaporized odor stimuli is delivered for 2 s. To receive water reward, the mouse should lick at least once in every 0.5 s intervals when the CS + stimulus is presented. The animal should stay in the port for at least 0.5 s while the CS is presented, for the trial to be considered valid. The performance criterion was set in 85% of correct responses in two blocks within the session. In the first experiment, isoamyl acetate was used as the rewarded stimulus or CS+, while mineral oil was used as CS-. Mice who did not reach criteria on the first session repeated the test the following day. All mice that reached criteria (in 1 or 2 days) repeated the task 5 weeks later to compare long-term olfactory memory between WT and *Fmr1* KO groups. The second experiment involved phenyl acetate as CS + and 2-butanone CS-. The test was repeated the following day, with a reversion of the hedonic values used on the previous day: 2-butanone was the CS + stimulus and phenyl acetate the CS-.

The third experiment involved the discrimination between two complex odorant mixtures: 10 C, who consists in 10 different monomolecular odorants diluted in mineral oil, as CS+; and 10 C-1, consisting of 9 of the 10 odorants used in 10 C in mineral oil, as CS-. The test was performed at 0,16% v/v and 1,6% v/v. Mice who did not reach criterium on the first session repeated the test the following day.

### Odorant preparation and delivery

All the odorants used in the experiments were purchased in Sigma-Aldrich. The solutions prepared were stored in glass vials and connected to a Slotnick-style 8-channel pinch-valve olfactometer [23]. The experiments were controlled by custom written MatLab scripts. Isoamyl acetate, phenyl acetate and 2-butanone were diluted to 1% in 10 ml of mineral oil. The complex mixture 10 C was prepared with isoamyl acetate, ethyl valerate, 5-methyl-2-hexanone, isopropyl benzene, 1,7-octadiene, 2-heptanone, heptanal, 4-methyl-3-penten-2-one, 1-pentanol, and nonane. For the 10 C-1 mixture nonane was excluded. The concentrations of each odorant used in these mixtures were extracted from a previous work [24]. Taking in consideration that Barnes et al. used rats in their experiments, and that mice were not motivated to be trained using those concentrations (as if they were repelled by the smell) we adjusted the concentrations to 1% and 10% of those in Barnes et al., corresponding to 0,16% v/v and 1,6% v/v respectively.

### Electrophysiology

*Slice preparation*: WT and *Fmr1* KO male mice between 30 and 41 postnatal days were anaesthetized using isoflurane and decapitated after ensuring absence of tail pinching reflex. The brain was quickly extracted and submerged in ice-cold cutting artificial cerebrospinal fluid (cACSF) solution, (in mM): 125 NaCl, 25 NaHCO_3_, 1.25 NaH_2_PO_4_, 3 KCl, 10 glucose, 0.5 CaCl_2_ and 6 MgCl_2_, at pH 7.3. Coronal slices of 300 μm thickness were obtained using a vibratome (1000 plus, Vibratome^®^). Slices were left to recover for 1 h at 37 °C in a submerged chamber containing regular ACSF solution, (in mM): 125 NaCl, 25 NaHCO_3_, 1.25 NaH_2_PO_4_, 3 KCl, 25 glucose, 1 CaCl_2_, 2 MgCl_2_, pH 7.25, 307 mOsm, supplemented with 2 mM ascorbic acid and 3 mM Na-pyruvate and equilibrated with 95% O_2_ and 5% CO_2_.

Current-clamp and voltage-clamp recordings of PC layer II neurons were done using an Axopatch-1D amplifier (Molecular Devices), under visual guidance by a Nikon Eclipse E600FN microscope and data was sampled at 20 kHz. The recording electrode consisted of a borosilicate capillary elongated using a puller (Sutter P87), filled with internal solution and resistance between 3 and 6 MΩ. Experiments were conducted at 30–34 °C and the.

recording chamber was continuously perfused with oxygenated ACSF (1–2 ml/min).

For current clamp experiments the internal solution contained (in mM): 123 K-gluconate, 10 KCl, 4 glucose, 1 EGTA, 10 HEPES, 2 Na-ATP, 0.2 Na-GTP, 10 Na-phosphocreatine, 0.1 CaCl_2_, 1 MgCl_2_, pH 7.35, 291 mOsm. Liquid junction potential (LJP) was 15 mV and was corrected offline afterwards during data analysis.

The whole-cell configuration was attained in voltage-clamp mode. For voltage-clamp experiments, two different internal solutions were used to record spontaneous excitatory post synaptic currents (sEPSC) and spontaneous inhibitory post synaptic currents (sIPSC). The internal solution for sEPSC recordings contained (in mM): 115 Cs-methanesulfonate, 20 CsCl, 0.6 EGTA, 10 HEPES, 4 Na_2_-ATP, 0.4 Na-GTP, 10 Na-phosphocreatine, 2.5 MgCl_2_, pH 7.25, 300 mOsm. Liquid junction potential was 7 mV and was corrected afterwards. For sIPSC recordings, the internal solution contained (in mM): 140 CsCl, 1 EGTA, 6 KCl, 4 NaCl, 10 HEPES, 2 MgCl_2_, 4 Na-ATP, 0.4 Na-GTP, pH 7.25, 300 mOsm. LJP for this solution was not directly measured but calculated using Clampex software. It was ~ 4 mV and was corrected offline afterwards. Voltage-clamp recordings of spontaneous synaptic currents were conducted at an uncorrected holding potential of -60 mV, in the presence of the GABA-A receptor inhibitor picrotoxin (PTX; 100 µM), for sEPSC recordings, and the AMPA receptor antagonist CNQX (20 µM), for sIPCS recordings. Series resistance was not compensated but it was continually monitored using a 200 ms, -5 mV pulse at the end of each sweep.

### Analysis of electrophysiological data

Data were analyzed using custom algorithms developed in IgorPro software (Wavemetrics, Oregon USA) supplemented with the Neuromatic v3.0 package. Series resistance was calculated from the initial peak current elicited by current elicited by 5 hyperpolarizing steps of 1mV each. Cells displaying more than 30 MΩ or a variation higher than 30% during the experiment, were discarded. Using the same protocol, membrane capacitance was calculated as the slope of the charge-voltage plot (C^m^ = Q/V), where the charge was obtained by integrating the transient capacitive current.

Resting membrane potential was measured shortly after attaining the *whole-cell* configuration in zero current mode. Input resistance was measured as the slope of the voltage-current relationship at the end of 500 ms hyperpolarizing current pulses ranging from − 60 to -20 pA, with 10 pA increments, always starting from an initial voltage of -70 ± 2.5 mV (-85 ± 2.5 mV, after LJP correction), adjusted from cell to cell by constant current injection. Membrane time constant was determined by adjusting a single exponential to the early voltage response to 100 ms current pulses of -20 pA (average of 10 traces). The sag ratio was evaluated by application of hyperpolarizing pulses of 500 ms starting from − 120 pA to -510 pA in increments of -30 pA and was calculated as the peak of voltage deflection divided by the steady state voltage.

Plots of spiking frequency versus current (F-I curves) were constructed by applying 500 ms pulses starting from 25 pA to up to 1 nA, in increments of 50 pA. To avoid the influence of the different resting potentials on Na^+^ channel basal inactivation, all experiments started from ∼ -85 mV. The rate of change of the firing frequency with increasing current (gain) was calculated as the slope of the linear region of the F-I curve by linear regression (typically considering the first 3–4 non-zero points). The rheobase was defined as the amplitude of the minimum current pulse eliciting at least one action potential. Spike threshold, amplitude, overshoot and half-width were obtained from this first action potential elicited using a similar protocol, but with increments of 5 pA. Action potential threshold was calculated from the phase plot (dV/dt versus V) and corresponded to the voltage of the first point where dV/dt > 10 mV/ms [25, 26].

Spontaneous synaptic events were detected using a custom algorithm of Igor Pro software based on the event detection tool of Neuromatic 3.0 plugin. Recordings were first smoothed using binomial smoothing of 25 coefficients. To select synaptic events, both amplitude and kinetics criteria were used. First, putative events were selected by amplitude, setting a 5 pA threshold relative to previous baseline, provided the amplitude was at least 2.5 times the standard deviation (SD) of the baseline. Event kinetics was characterized by the rise and decay times. Rise time was calculated from 10 to 90% of the rising phase and decay time was obtained by fitting a single exponential to the decay phase. Events with decay time less than 1 ms or shorter than the rise time, were also discarded. Inter-event intervals were calculated as the period between consecutive event onset times, and the average frequency per cell was calculated as the inverse of their average inter-event interval.

To compare cumulative probability distributions (CPDs) between WT and *Fmr1* KO groups, we employed a bootstrapping approach to address potential biases caused by imbalances in sample sizes per cell. Random resampling with replacement was performed to generate datasets with equal representation across cells, where the number of resampled data points per cell was equal to the mean number of events recorded across all cells (375 for sEPSCs and 1000 for sIPSCs). CPDs were computed for each resampled dataset and averaged over 10,000 iterations to ensure robustness.

### Statistical analysis

For behavioral experiments, the percentage of correct answers was calculated for each block and a mixed-effects analysis was used to compare the performance of both groups in the different go-no go tasks. Bonferroni corrections were applied to ensure statistical robustness of multiple comparisons in each block. Additionally, a complementary method was used to evaluate the efficiency of WT and *Fmr1* KO mice throughout the tests: the Receiver Operating Characteristics (ROC) analysis allows to graphically represent the accuracy of a binary classifier. In this work, the ROC analysis was used to determine the accuracy of WT and *Fmr1* KO mice (the classifiers) in discriminating two different stimuli (CS + and CS-). The ROC graph is created by plotting the true positive rate (TP, licking to CS+) against the false positive rate (FP, licking to CS-). The diagonal, corresponding to an area under the curve (AUC-ROC) of 0.5, represents random behavior, and the bigger the AUC-ROC, the better the classifier. AUC-ROC was calculated for the first and last of 60 trials conducted in each session, and a previously described statistical method was used to compare and evaluate the differences between the WT and the *Fmr1* KO ROC curves [27].

For electrophysiological data statistical analyses were done using Prism 7 software (GraphPad Software, USA) and data is presented as the mean ± standard deviation (SD), whereas graphs show the mean and standard error of the mean (S.E.M). For the analysis of spontaneous synaptic currents, statistical significance was assessed using a permutation-based Kolmogorov-Smirnov (KS) test, where the distribution of KS statistics obtained from all resampled datasets was compared against a null distribution of KS statistics generated by randomly shuffling the data. For each comparison, p-values were estimated as the proportion of shuffled KS statistics greater than or equal to the observed KS statistics. The analysis was implemented using custom code written in Python 3.8.

## Results

### Discrimination of binary and complex odor mixtures

**Fig. 1:**
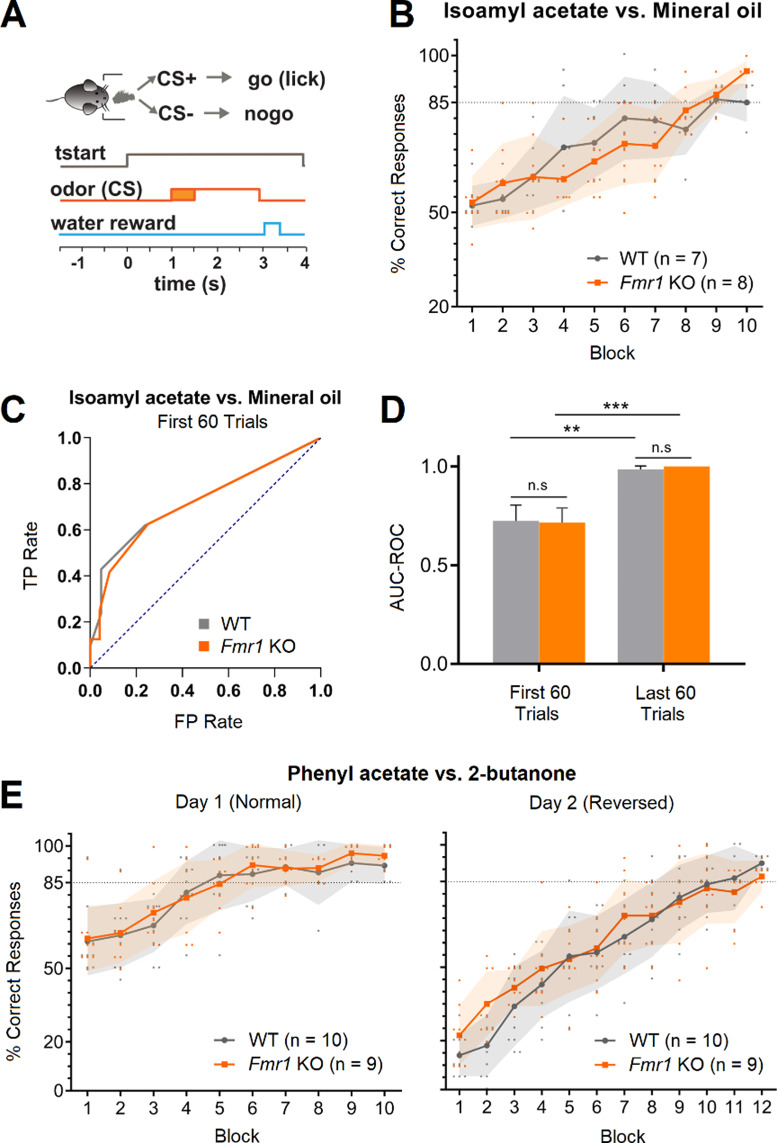
*Fmr1* KO mice have similar olfactory learning capabilities compared to WT mice but show a higher detection threshold for nonane. **(A)** Diagram of the go-no go behavioral task. Once the animal placed its snout in the odor port the olfactory stimulus was delivered for two seconds. The animal was required to stay at least for 500ms in the odor port after the stimulus was delivered for a trial to be considered as valid. If the animal licked a water tube during CS + presentation, water was delivered as a reward. **(B)** Behavioral responses to isoamyl acetate (CS+) vs. mineral oil (CS-). WT and *Fmr1* KO mice show similar olfactory learning. Mice who did not reach criterium on the first day repeated the test the following day (Fig. S1). **(C)** Receiver operating characteristics (ROC) analysis. The ROC graph depicts relative tradeoffs between benefits (true positives: TP, licking to CS+) and costs (false positives: FP, licking to CS-). The diagonal (blue dotted line) represents random behavior, and the bigger the area under the curve (AUC-ROC) the better the classifier. **(D)** AUC-ROC summary of the first and last 60 trials of the isoamyl acetate vs. mineral oil task. **(E)** Behavioral responses to phenyl acetate vs. 2-butanone. On the first day, phenyl acetate served as the CS + and 2-butanone as the CS-. The following day, the hedonic values of the two odorants were reversed. In B and E, shaded area represents mean ± standard deviation (SD). In D, bars represent the standard error (SEM; *p < 0,05, **p < 0,01, ***p < 0,001, ****p < 0,0001)

**Fig. 2:**
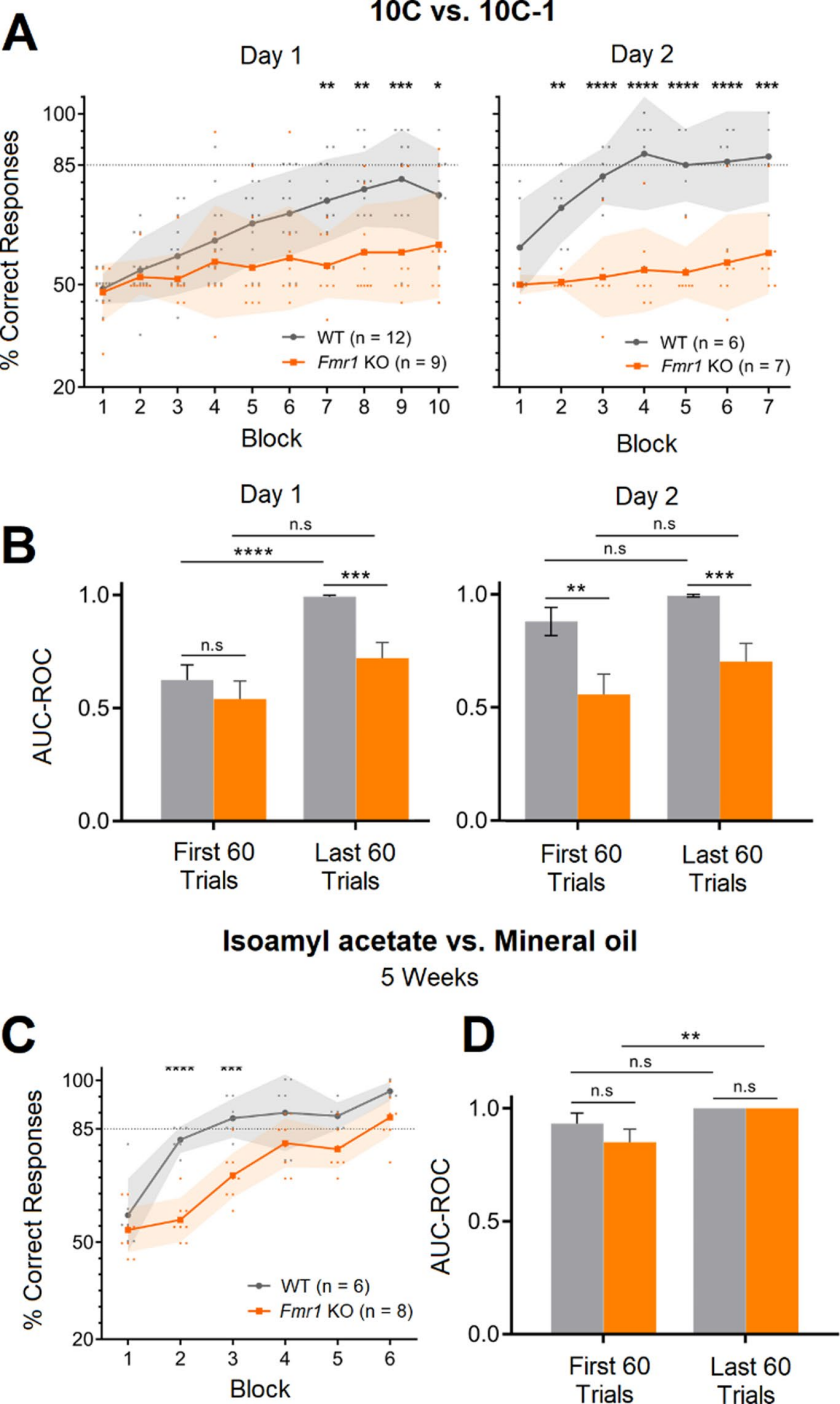
*Fmr1* KO mice show impairments in the discrimination of complex odor mixtures. **(A)** Behavioral responses of the go/no-go task involving the discrimination of two complex odor mixtures: 10 C (CS+) is a mixture of 10 monomolecular odorants diluted in mineral oil, while 10 C-1 (CS-) is composed of 9 of the 10 odorants used for 10 C. Mice who did not reach the criterium on the first day repeated the test the following day. Compared to WT mice, *Fmr1* KO shows deficiencies in correctly discriminating 10 C from 10 C-1. **(B)** AUC-ROC summary of the first and last 60 trials of each session of 10 C vs. 10 C-1 task. **(C)** Behavioral responses to isoamyl acetate (CS+) vs. mineral oil (CS-), 5 weeks later. Only the mice that previously reached criterium on this task were allowed to perform the test in this instance. Compared to WT mice, *Fmr1* KO shows a worse performance in the first blocks of the session, suggesting deficiencies in correctly remembering previously learned olfactory tasks. **(D)** AUC-ROC summary of the first and last 60 trials of the session. In A, shaded area represents mean ± standard deviation (SD). In B and D, bars represent the standard error (SEM; *p < 0,05, **p < 0,01, ***p < 0,001, ****p < 0,0001)

The ability of the *Fmr1* KO to discriminate olfactory stimuli was assessed using an olfactory go-no go behavioral task. In this task, thirsty animals are trained to lick into a metal tube located below the odor port in response to a rewarded stimulus (CS+) and refrain from licking in response to an unrewarded stimulus (CS-). If the task is performed correctly, water is given as a reward (Fig. 1A). Animals perform one session per day consisting in 200 trials or 10 blocks (20 trials are randomly assigned to CS + and CS- per block and averaged together for visualization and analysis purposes) [23]. Licking in response to CS+ (hit) and refrain from licking in response to CS- (correct rejection) are considered correct responses. On the other hand, licking in response to CS- (false alarm) and not licking following CS+ (miss) are considered incorrect responses. We first trained the animals in a binary discrimination task to study whether *Fmr1* KO mice were able to associate an olfactory stimulus with a reward. We used mineral oil (CS-) versus isoamyl acetate diluted in mineral oil (CS+) [23], which is classified as an “easy” task for mice (Nunez-Parra, 2020) since both of them elicit dramatically different activation maps in the glomerular layer of the olfactory bulb [28]. We found that *Fmr1* KO mice can discriminate between mineral oil and isoamyl acetate, quickly reaching above chance criteria (85% or more of correct responses in three consecutive blocks) in only one session (Fig. 1B). We also did not find differences in the number of blocks *Fmr1* KO mice performed per session indicating proper motivation response. In a similar study, the Larson group [12] studied *Fmr1* KO mice using a two alternative olfactory forced choice paradigm and found that *Fmr1* KO could learn to associate an olfactory cue with a reward, but also observed that the KO made more errors and learn slower compared to WT. Therefore, to compare the performance of WT and *Fmr1* KO we performed a receiver operating characteristics (ROC) analysis. The ROC curve depicts the relative tradeoffs between the true positive rate: TP rate, probability of licking to CS+, and false positive rate: FP rate, the probability of licking to CS-. The diagonal (blue dotted line) represents random behavior and the bigger the area under the curve (AUC-ROC), that is the higher left the curve goes, the better the classifier and the performance (Fig. 1C). We found that there is a significant increase in the AUC-ROC value during the first 60 trials of the session (when the animal is learning to associate the CS with the reward) compared to random behavior, and that there is an even better performance during the last 60 trials of the task where animals reach proficiency (Fig. 1D; WT AUC-ROC: 0.73 ± 0.08 in the first 60 trials and 0.98 ± 0.02 in the last 60 trials, *n* = 7, Hanley-McNeil: *p* = 0.0015; *Fmr1* KO AUC-ROC: 0.72 ± 0.08 in the first 60 trials and 1 ± 0 in the last 60 trials, *n* = 8, Hanley-McNeil: *p* = 0.0002. AUC-ROC values are presented as Mean ± SEM). However, we did not find any differences between strains, suggesting that WT and *Fmr1* KO mice performance in the go-no go task do not differ (WT vs. *Fmr1* KO AUC-ROC in the first 60 trials, Hanley-McNeil: *p* = 0.93; WT vs. *Fmr1* KO AUC-ROC in the last 60 trials, Hanley-McNeil: *p* = 0.38). To confirm our results, when animals were proficiently discriminating mineral oil from isoamylacetate, we trained them to discriminate an additional pair of odorants: 2-butanone (CS-) and phenylacetate (CS+). We found that both, proficient WT and *Fmr1* KO animals, were faster at discriminating novel olfactory stimuli, with some of them reaching more than 85% of correct responses during the second block of the session (Fig. 1E, left; *n* = 10 WT and 9 *Fmr1* KO, Mixed-effects analysis with post hoc Bonferroni’s multiple comparison test between WT and *Fmr1* KO: *p* > 0.05 in all blocks of Day 1). Moreover, WT and *Fmr1* KO were able to reverse the hedonic value of the stimuli and discriminating 2-butanone as the (CS+) and phenylacetate as the (CS-). Note that they start close to 20% of correct responses and in one session they reach criteria (Fig. 1E, right; *n* = 10 WT and 9 *Fmr1* KO, Mixed-effects analysis with post hoc Bonferroni’s multiple comparison test between WT and *Fmr1* KO: *p* > 0.05 in all blocks of Day 2). Next, we evaluated whether *Fmr1* KO animals were able to discriminate complex odor mixtures. To do this, we used a mixture of 10 odorants as the rewarded stimulus (10 C, CS+; isoamyl acetate, ethyl valerate, 5-methyl-2-hexanone, isopropyl benzene, 1,7-octadiene, 2-heptanone, heptanal, 4-methyl-3-penten-2-one, 1-pentanol, and nonane) versus the same mixture but lacking nonane as the unrewarded stimuli (10–1C, CS-). Perceptual discrimination of these two particularly similar (and complex) olfactory stimuli has been suggested to be a “hard task” as the stimuli will induced overlapped neuronal activation patterns [24]. Compared to monomolecular odorants, we found that this task was harder for animals to complete and took WT mice on average two sessions to reach criteria (Fig. 2A). Importantly, *Fmr1* KO were not capable of reaching criteria even after two training sessions, suggesting that olfactory processing is altered in this animal model(Fig. 2A; Day 1: *n* = 12 WT and 9 *Fmr1* KO, Mixed-effects analysis with post hoc Bonferroni’s multiple comparison test between WT and *Fmr1* KO: *p* < 0.05 on blocks 7 to 10; Day 2: *n* = 6 WT and 7 *Fmr1* KO, Mixed-effects analysis with post hoc Bonferroni’s multiple comparison test between WT and *Fmr1* KO: *p* < 0.01 on blocks 2 to 7). Consistent with these results, the AUC-ROC analysis also showed differences between the WT and the *Fmr1* KO when the last 60 trials where compared during Day1 and Day2 (Fig. 2B; WT AUC-ROC = 0.99 ± 0.007 and *Fmr1* KO AUC-ROC = 0.72 ± 0.070 in the last 60 trials of Day 1, Hanley-McNeil: *p* = 0.0001; WT AUC-ROC = 0.99 ± 0.005 and *Fmr1* KO AUC-ROC = 0.70 ± 0.08 in the last 60 trials of Day 2, Hanley-McNeil: *p* = 0.0003. AUC-ROC values are presented as Mean ± SEM).

These results suggested that other cognitive functions involving the PC could also be altered in the *Fmr1* KO. Therefore, we decided to test long-term memory in these mice. To do this we evaluated whether mice could still distinguish between isoamyl acetate and mineral oil five weeks after they became proficient in the go-no go task. In this case, we found differences between groups. Both started the test at chance levels, but the WT animals rapidly reached criteria suggesting that they remember the task (Fig. 2C). On the contrary, the *Fmr1* KO animals showed similar behavior as the first day of training in the go- no go task (Fig. 1B), slowly reaching criteria during the first session (Fig. 2C; *n* = 6 WT and 8 *Fmr1* KO, Mixed-effects analysis with post hoc Bonferroni’s multiple comparison test between WT and *Fmr1* KO: *p* < 0.001 on blocks 2 and 3). The AUC-ROC analysis confirmed that there are differences in the performance of KO when the first and last 60 trials were compared (Fig. 2D; WT AUC-ROC = 0,93 ± 0,05 and *Fmr1* KO AUC-ROC = 0.85 ± 0.06 in the first 60 trials, Hanley-McNeil: *p* = 0.28; WT AUC-ROC = 1 ± 0 and *Fmr1* KO AUC-ROC = 1 ± 0 in the last 60 trials, first 60 vs. last 60 trials in the *Fmr1* KO, Hanley-McNeil: *p* = 0.009; AUC-ROC values are presented as Mean ± SEM).

Taken together our results show that neuronal processes such as olfactory discrimination and long-term memory are deficient in *Fmr1* KO suggesting that physiological alteration in the PC could in part underpin these observations.

### Layer II principal neurons of the piriform cortex display enhanced excitability

**Fig. 3:**
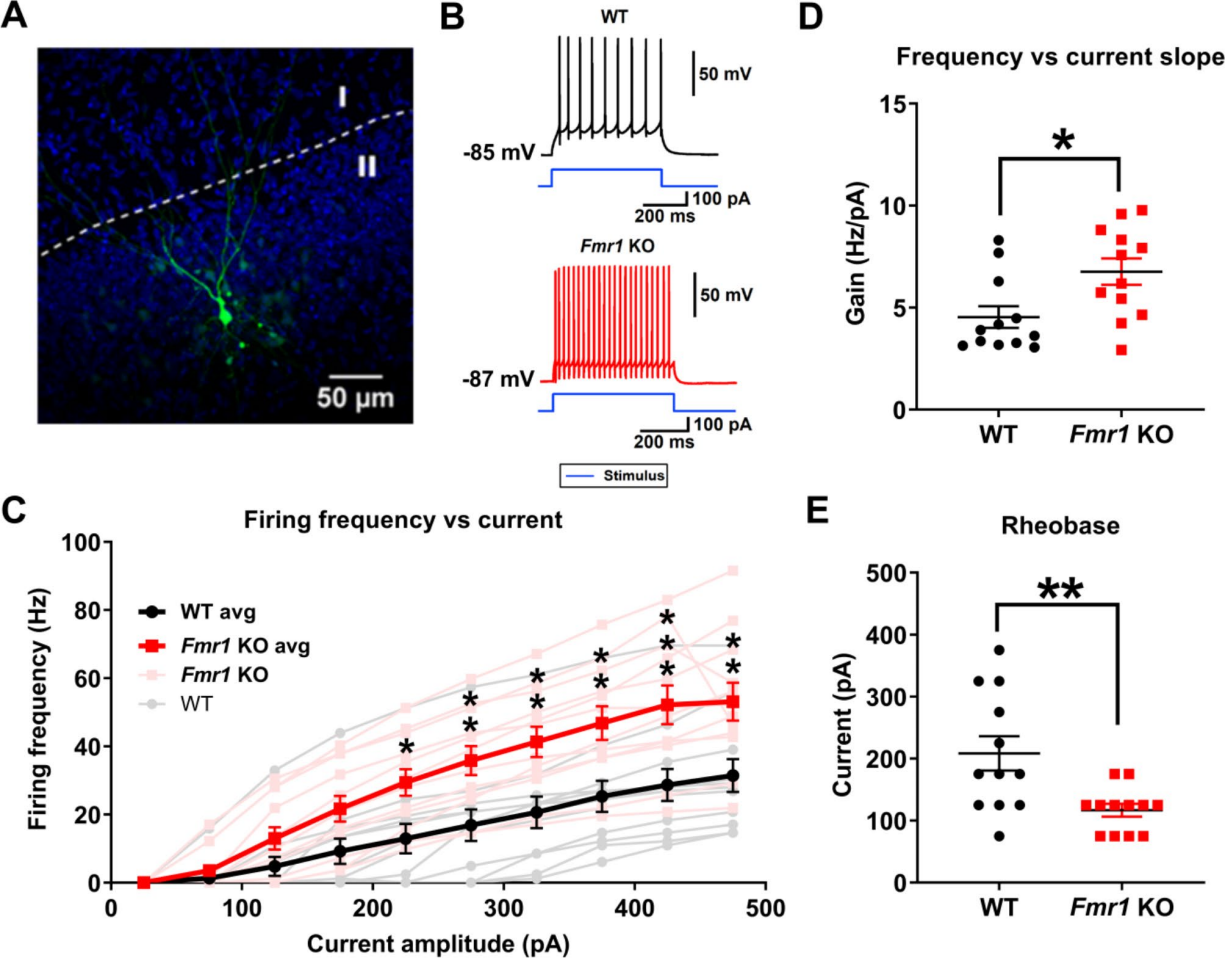
Whole cell patch clamp recordings showed that Piriform Cortex (PC) layer II neurons from *Fmr* 1 KO mice are hyperexcitated compared to WT control. **(A)** Confocal fluorescent microphotography of a biocytin-labeled neuron recorded in layer II of the PC from a brain slice of a *Fmr1* KO mouse. The nuclei from surrounding cells are stained with DAPI. **(B)** Representative whole-cell current-clamp recordings in response to a current pulse of 125 pA (blue traces) of neurons from WT (black) and *Fmr1* KO (red) mice. **(C)** Firing frequency in response to increasing current steps from WT (black circles) and *Fmr1* KO (red squares) neurons, starting from a holding voltage of -85 ± 2.5 mV. For both groups, *n* = 12. Light colors represent individual cells while bold colors show the average firing frequency vs. current curves of WT and *Fmr1* KO cells. Two-way ANOVA results: strain factor *p* < 0.0001; current factor *p* < 0.0001; interaction: *p* = 0.0212. Sidak’s multiple comparison (*: *p* < 0.05, **: *p* < 0.01; ***: *p* < 0.0001). **(D)** Rheobase of WT and *Fmr1* KO neurons obtained from the intercept with the X axis of each firing frequency curve shown in B. **(E)** Firing frequency slope of the linear segment of each curve shown in B. Here, and in all following figures, middle horizontal line and bars correspond to the mean and S.E.M respectively. All the upcoming figures follow the same color scheme used here, where black represents data from WT cells and red from *Fmr1* KO cells

Because several cortical regions have been reported to exhibit a hyperexcitable phenotype in the *Fmr1* KO mice model [4], we examined if the olfactory behavioral alterations seen in the *Fmr1* KO might be related to differences in PC cellular excitability. To do this, we performed whole-cell patch clamp recordings from PC layer II principal neurons to evaluate active and passive membrane properties of this cell population. An example of a principal cell was confirmed by immunostaining of biocytin-filled recorded neurons (Fig. 3A). First, we assessed neuron excitability measuring the number of action potentials (AP) elicited in response to a series of current steps of increasing amplitude and equal duration (F-I curve, see Methods). Results from these experiments show that layer II principal cells from *Fmr1* KO mice exhibit a higher firing frequency response to similar current injections (Fig. 3B and C; *n* = 12 for WT and *Fmr1* KO; Two-way ANOVA: *p* = 0.02). The increment in AP frequency in the *Fmr1* KO group was detected for current stimuli as low as 225pA and gets stronger for larger current steps (Fig. 3C; Post hoc Sidak’s multiple comparison test *p* = 0.039). As a measure of the gain, we quantified the slope of the linear segment of the F-I curve for each recorded cell (see Methods). Our data indicate that layer II principal neurons from *Fmr1* KO display a significantly steeper F-I curve slope than WT neurons (Fig. 3D; WT: 4.54 ± 1.8 Hz/pA, *n* = 12; *Fmr1* KO: 6.76 ± 2.2 Hz/pA, *n* = 12; t-test, *p* = 0.014), further supporting the hypothesis of an increased principal neuron excitability. As an additional measure of excitability, we evaluated the rheobase, which is the minimum current needed to elicit at least one AP. We found that *Fmr1* KO layer II principal cells display a lower rheobase compared to WT neurons (Fig. 3E; WT: 208.3 ± 96 pA, *n* = 12; *Fmr1* KO: 116.7 ± 36 pA, *n* = 12; Welch’st-test, *p* = 0.008), indicating that less current is needed to reach AP threshold.

**Fig. 4:**
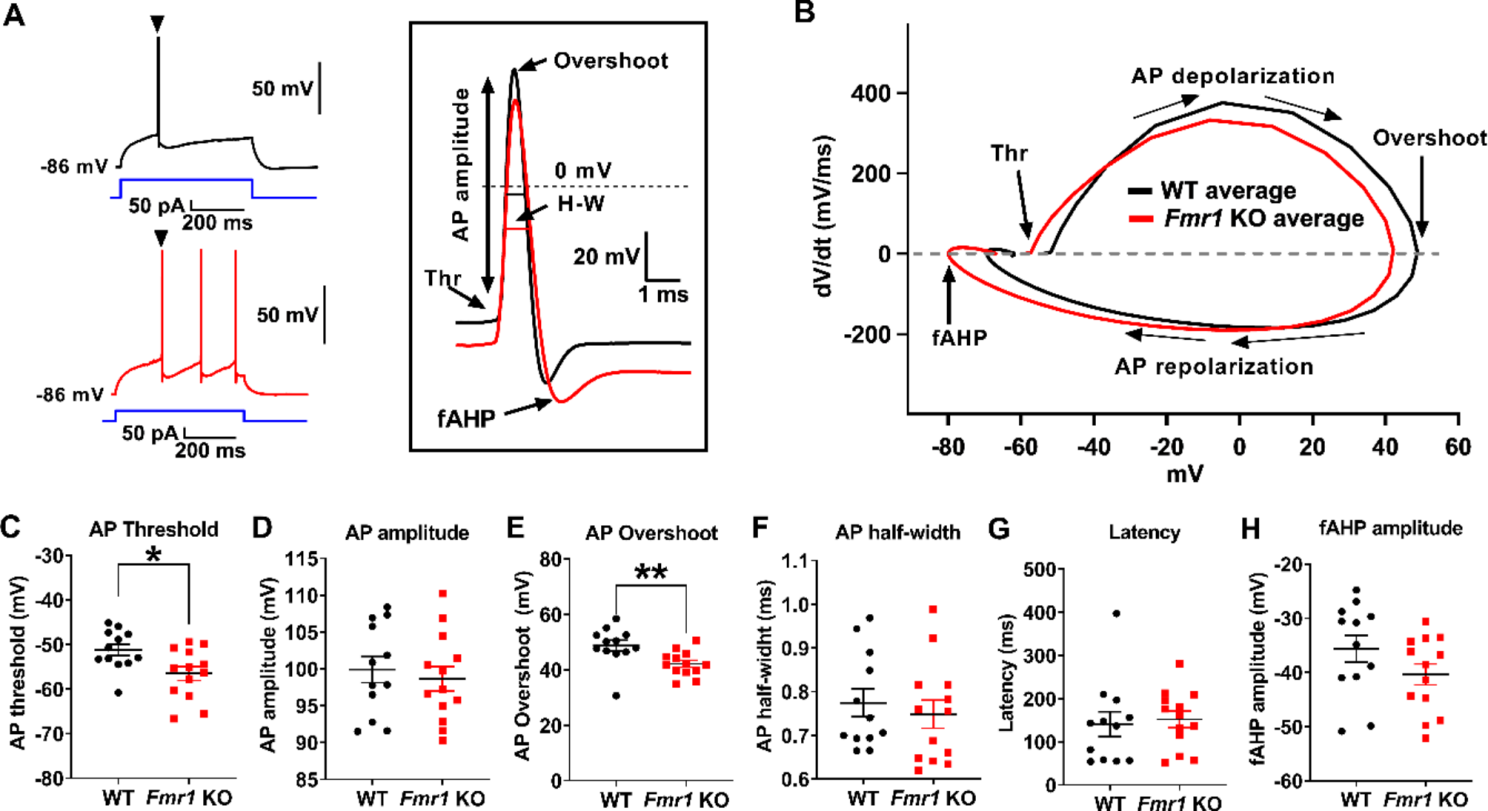
*Fmr1* KO pyramidal neurons have lower threshold for action potential generation compared to the WT. **(A)** Representative traces of voltage responses to depolarizing current steps of 5 pA until the appearance of an action potential, triangles indicate the first spike. Inset shows examples of first spikes aligned by peak, highlighting relevant properties: threshold (Thr), amplitude, overshoot, half-width (H-W) and fast after hyperpolarization (fAHP) are indicated. **(B)** Average phase-plot of the first evoked spike of each cell. Arrows illustrate the relevant information that can be obtained from the plot. **(C)** Action potential (AP) threshold determined as the voltage where dV/dt > 10 mV/ms. **(D)** AP amplitude determined from threshold to peak. **(E)** AP overshoot form 0 mV. **(F)** AP half-width. **(G)** Latency to reach threshold from onset of the stimulus. **(H)** fAHP amplitude calculated as the minimum voltage reached minus threshold (*: *p* < 0.05; **: *p* < 0.01)

We next evaluated whether these alterations in excitability are associated to changes in active membrane properties using a protocol of small current steps (see details in Methods) to examine AP threshold, half width, amplitude and overshoot, as well as fast afterhyperpolarization amplitude (fAHP) (Fig. 4). To establish an initial comparison between APs from each mice line, phase plots were constructed to allow easier visualization and analysis of the AP trajectory and properties (Fig. 4B). Thus, AP threshold was calculated taking the voltage point for which dV/dt surpassed 10 mV/ms [25, 26], showing that *Fmr1* KO cells have more hyperpolarized AP thresholds than WT neurons (Fig. 4C; WT: -51.2 ± 4.5 mV, *n* = 12; *Fmr1* KO: -56.5 ± 5.7 mV, *n* = 13; t-test, *p* = 0.0165), which is consistent with the previously described increased excitability. We also found that the AP overshoot, corresponding to the voltage value at the peak, was different between groups (Fig. 4E; WT: 48.7 ± 6.8 mV, *n* = 12; *Fmr1* KO: 42.2 ± 4.5 mV, *n* = 13; t-test, *p* = 0.009). Interestingly, there was no difference in AP amplitude measured from the threshold to the peak voltage (Fig. 4D; WT: 99.93 ± 6.23 mV, *n* = 12; *Fmr1* KO: 98.67 ± 5.99 mV, *n* = 13; *p* = 0.612). On the other hand, we found no differences in AP half-width (Fig. 6G; WT: 0.77 ± 0.11ms, *n* = 12; *Fmr1* KO: 0.75 ± 0.12 ms, *n* = 13; *p* = 0.5721). While it would be tempting to interpret this observation as suggesting that conductances involved in repolarization are not altered, a diversity of voltage-gated Ca^2+^ currents and voltage- and Ca^2+^-dependent K^+^ channels could differentially fasten or retard this phase, such that changes in specific components could be masked (See Discussion). Moreover, the average phase plot in Fig. 4B shows a lower dV/dt for the AP rising phase (upstroke) in *Fmr1* KO neurons, which could also affect half-width.

The latency, defined here as the time for the cell to reach the first AP threshold from stimulus onset, was also not different (Figs. 4G and 141.1 ± 98.22 ms, *n* = 12; *Fmr1* KO: 152.2 ± 67.8 ms, *n* = 13; *p* = 0.7447). Lastly, we assessed the fast afterhyperpolarization (fAHP), a behavior of membrane potential after an AP is elicited. The presence of a fAHP has been associated to increase firing rates due to the removal of Na^+^ channel inactivation and the subsequent triggering of APs. We measured fast AHP (fAHP), which usually rely on fast inactivating Kv3 subfamily K^+^ channels [29] as well as large-conductance voltage- and Ca^2+^-dependent K^+^ channels (BK channels), promoting repetitive spiking. We found that fAHP, measured with respect to AP threshold (Fig. 4H), was not different between groups (WT: -35.6 ± 8.7 mV, *n* = 12; *Fmr1* KO: -40.4 ± 7.0 mV, *n* = 13; *p* = 0.1471). Altogether, the change in AP threshold suggests the involvement of voltage-dependent conductances in the hyperexcitable phenotype seen in *Fmr1* KO cells.

**Fig. 5:**
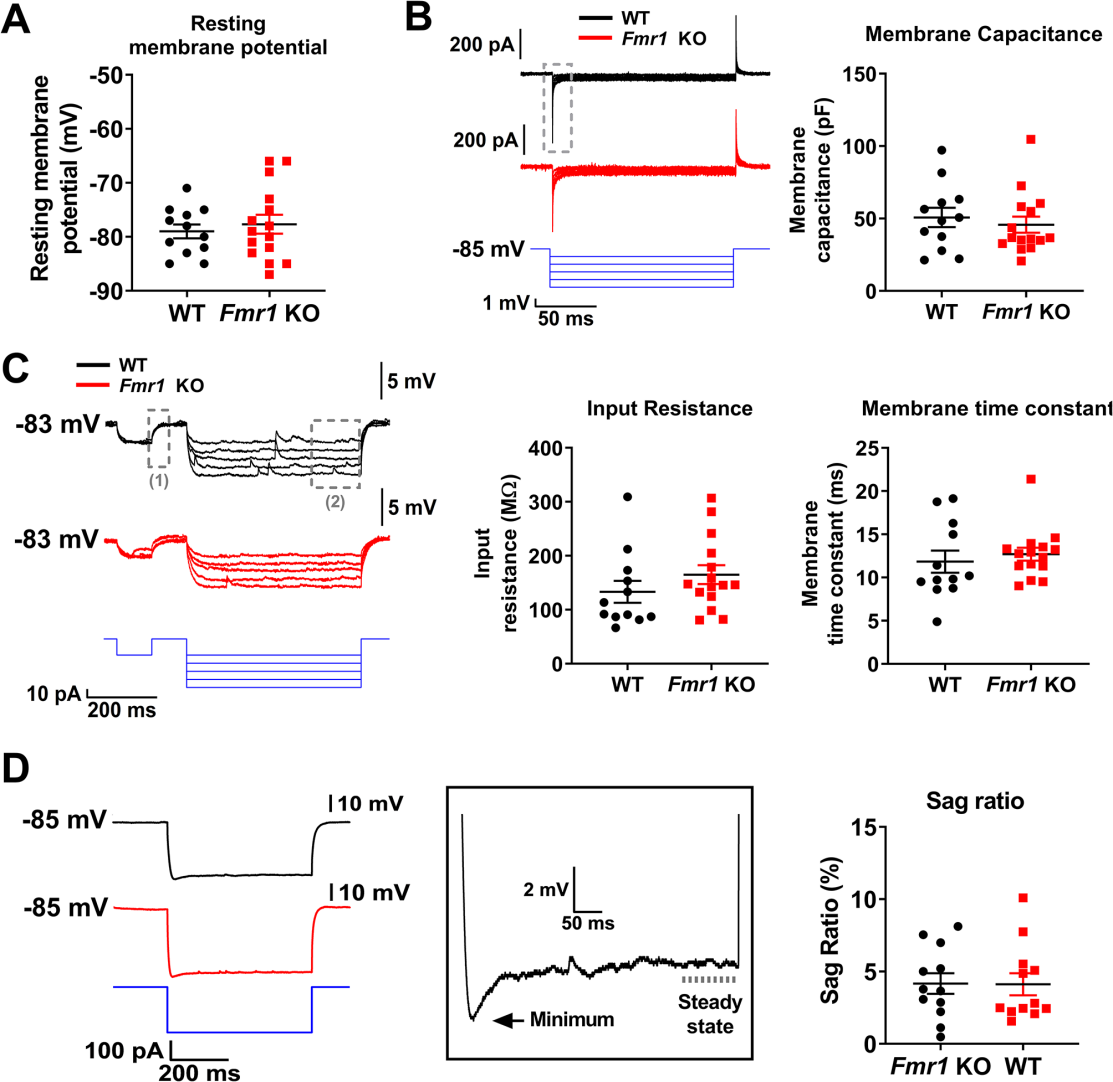
There are no differences in the passive membrane properties and sag ratio from layer II PC neurons in the *Fmr1* KO mice compared to WT. **(A)** Resting membrane potential measured briefly after attaining whole cell configuration in current-clamp mode I = 0. **(B)** Left: Sample traces of voltage clamp step protocol used to calculate the membrane capacitance (Cm). The dashed box shows the capacitive transient used to calculate Cm. Right: membrane capacitance for each cell. **(C)** Left: Sample traces from the protocol used for input resistance (Rin) and Tau (τ) calculation. Dashed boxes show the segment used for τ (1) and Rin (2) calculation as described in materials and methods. Middle: Rin for each cell. Right: Cm for each cell. **(D)** Left: Sample traces of the protocol used to calculate sag ratio. Inset shows zooming of one recording illustrating the presence of sag. Arrows indicate the minimum voltage reached and bar shows the steady state segment used for Sag ratio calculation. No differences between both groups’ means where found for all data shown

Finally, because hyperexcitability can arise from changes in cell passive properties involving leak channels modulation, expression and function [30–32], as well as cell size we also examined resting potential, membrane capacitance, input resistance and time constant in WT and *Fmr1* KO neurons. Briefly after attaining the whole-cell configuration, we switched to current clamp (I = 0) to estimate the resting membrane potential (Fig. 5A). We found no difference between WT (-79.0 ± 4.4 mV, *n* = 12) and *Fmr1* KO (-77.7 ± 6.8 mV, *n* = 15) animals (Welch’s t-test; *p* = 0.56). These values are consistent with previous reports on PC layer II pyramidal neurons from WT mice [33]. Similarly, membrane capacitance (C_m_), a correlate of somatic size, showed no differences between *Fmr1* KO and WT neurons (Fig. 5B; WT: 50.7 ± 23.1 pF, *n* = 12; *Fmr1* KO: 45.7 ± 21.5 pF, *n* = 15; Welch’s t-test; *p* = 0.57). Then, we evaluated the cell membrane resistance, which determines the amount of current needed to change the membrane potential, and the time constant (time needed to charge/discharge the membrane capacitor). Both parameters showed no differences between groups (Fig. 5C; WT = 133.3 ± 70.4 MΩ, *n* = 12; *Fmr1* KO = 164.9 ± 67.6 MΩ, *n* = 15, t-test. *p* = 0.25; and WT = 11.8 ± 4.4 ms, *n* = 12; *Fmr1* KO = 12.7 ± 2.9 ms, *n* = 15; t-test, *p* = 0.55, respectively).

**Fig. 6:**
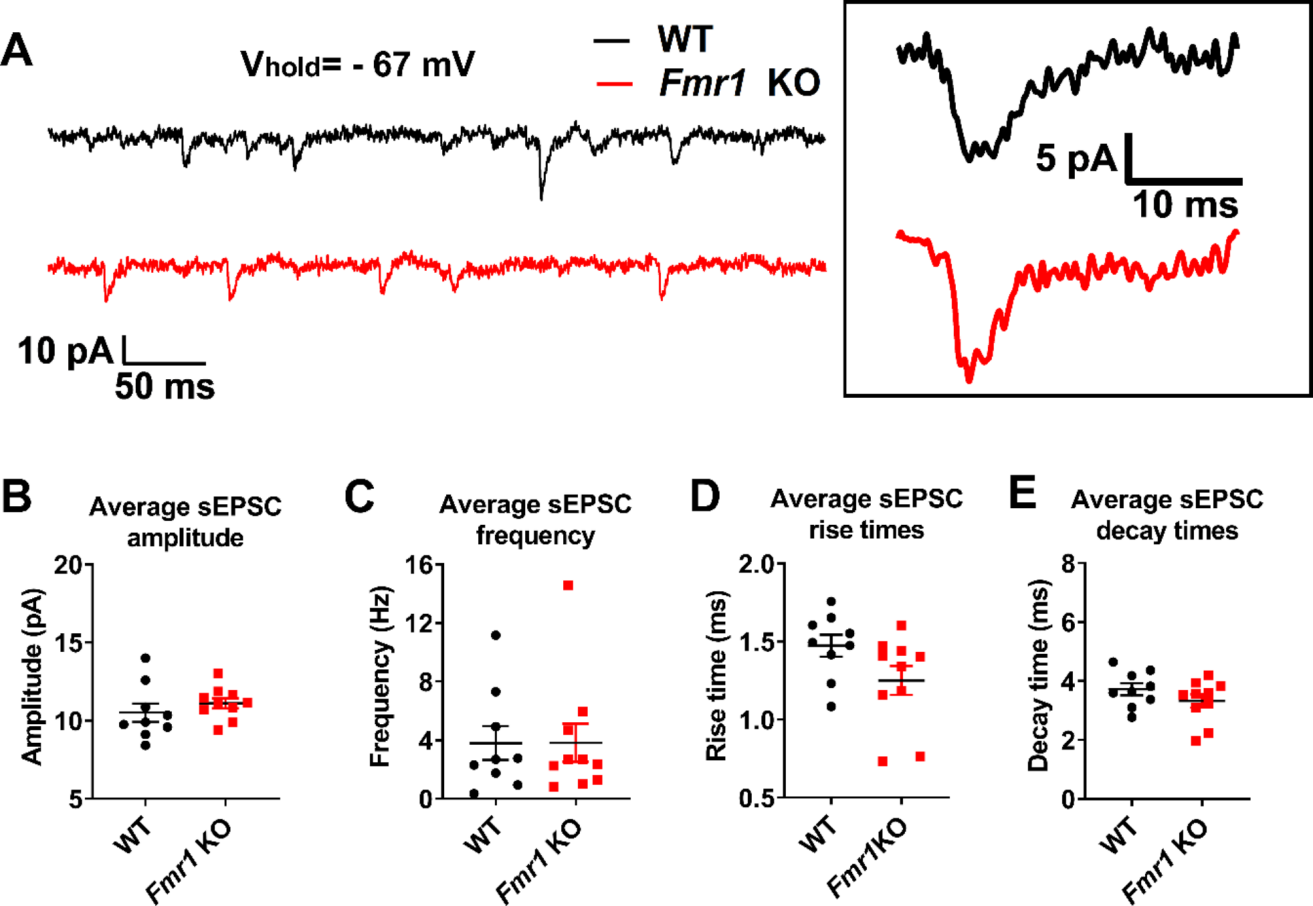
Spontaneous excitatory postsynaptic currents (sEPSC) recordings from *Fmr1* KO mice show similar average amplitude, frequency, and kinetics. **(A)** Representative sample traces of sEPCS recorded from *Fmr1* KO (red) and WT (black) neurons. Inset shows representative sEPSC events from *Fmr1* KO and WT cells. Graphs **(B)**, **(C)**, **(D)** and **(E)** show the per-cell average of sEPSC amplitude, frequency, rise time and decay time, respectively, for *Fmr1* KO and WT cells

While it is not a passive property, we included in this figure a quantification of the sag ratio as a measure of the presence of the hyperpolarization-dependent current, h-current, which can affect cell intrinsic excitability by reducing the effective input resistance. While h-current has been showed to be augmented in *Fmr1* KO in neurons of the somatosensory cortex [34], we found no alterations in sag ratio in PC neurons from *Fmr1* KO (Fig. 5D.2 ± 2.5%, *n* = 12; *Fmr1* KO: 4.1 ± 2.6% *n* = 12, Welch’s t-test *p* = 0.96) suggesting that this current does not contribute to the observed hyperexcitability. Altogether, passive membrane properties do not underlie the hyperexcitability phenotype observed in the layer II principal cells of the PC.

### Altered synaptic excitatory-inhibitory balance in the piriform cortex of the *Fmr1 KO* mice

**Fig. 7:**
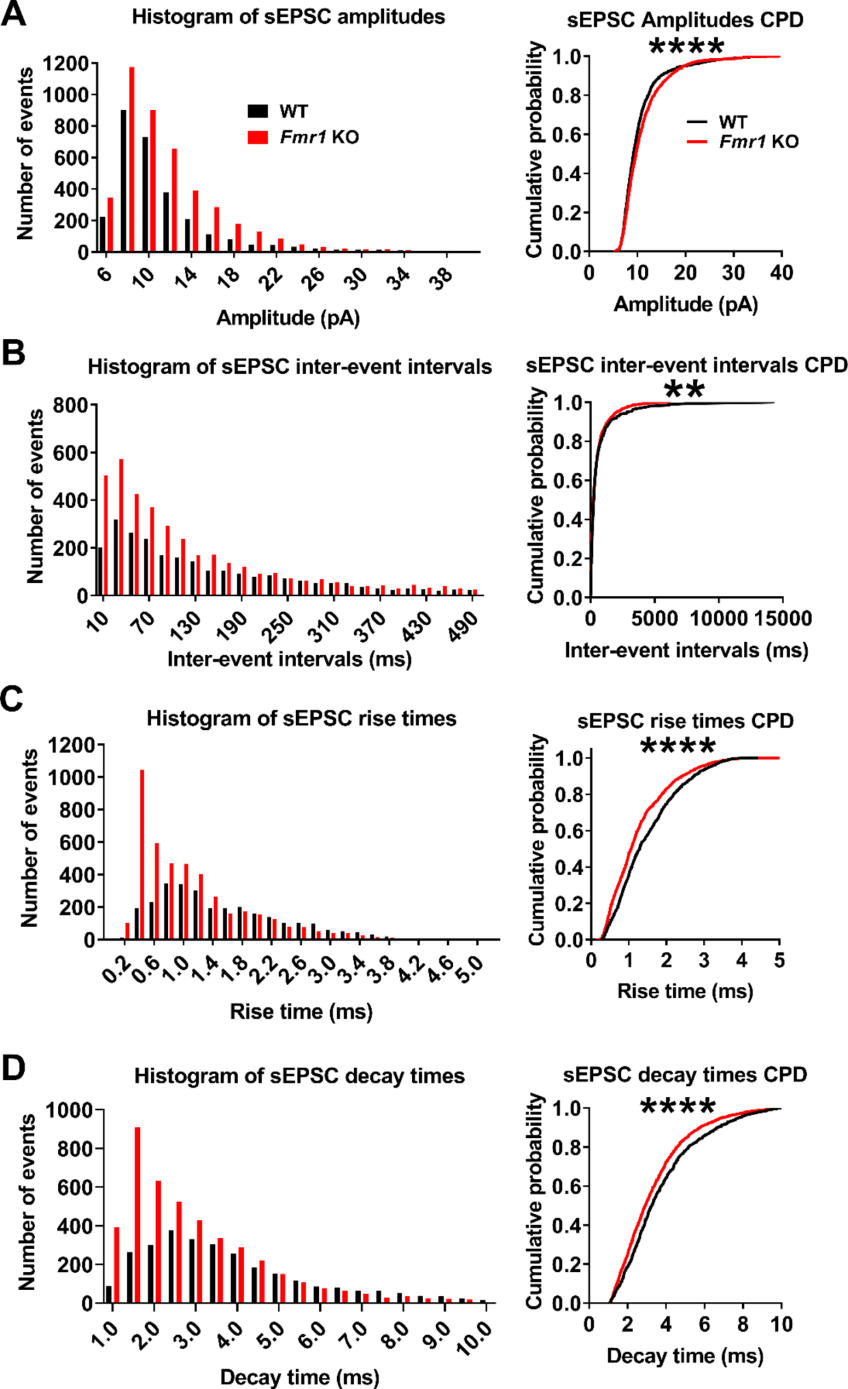
Distribution of sEPSC population´s amplitude, inter-event interval and kinetic parameters from *Fmr1* KO and WT neurons are different. **(A)** Left: Histogram of sEPSC amplitudes from all WT and all *Fmr1* KO cells (bin = 2 pA). Right: bootstrapped cumulative probability distribution (CPD) graph of sEPSC amplitudes. Graphs **(B)**, **(C)** and **(D)** show the same as in **(A)** but for inter-event intervals (bin = 50 ms), rise times (bin = 0.2 ms) and decay times (bin = 0.5 ms) respectively. Distributions are different between groups (**: *p* < 0.01; ****: *p* < 0.0001)

**Fig. 8:**
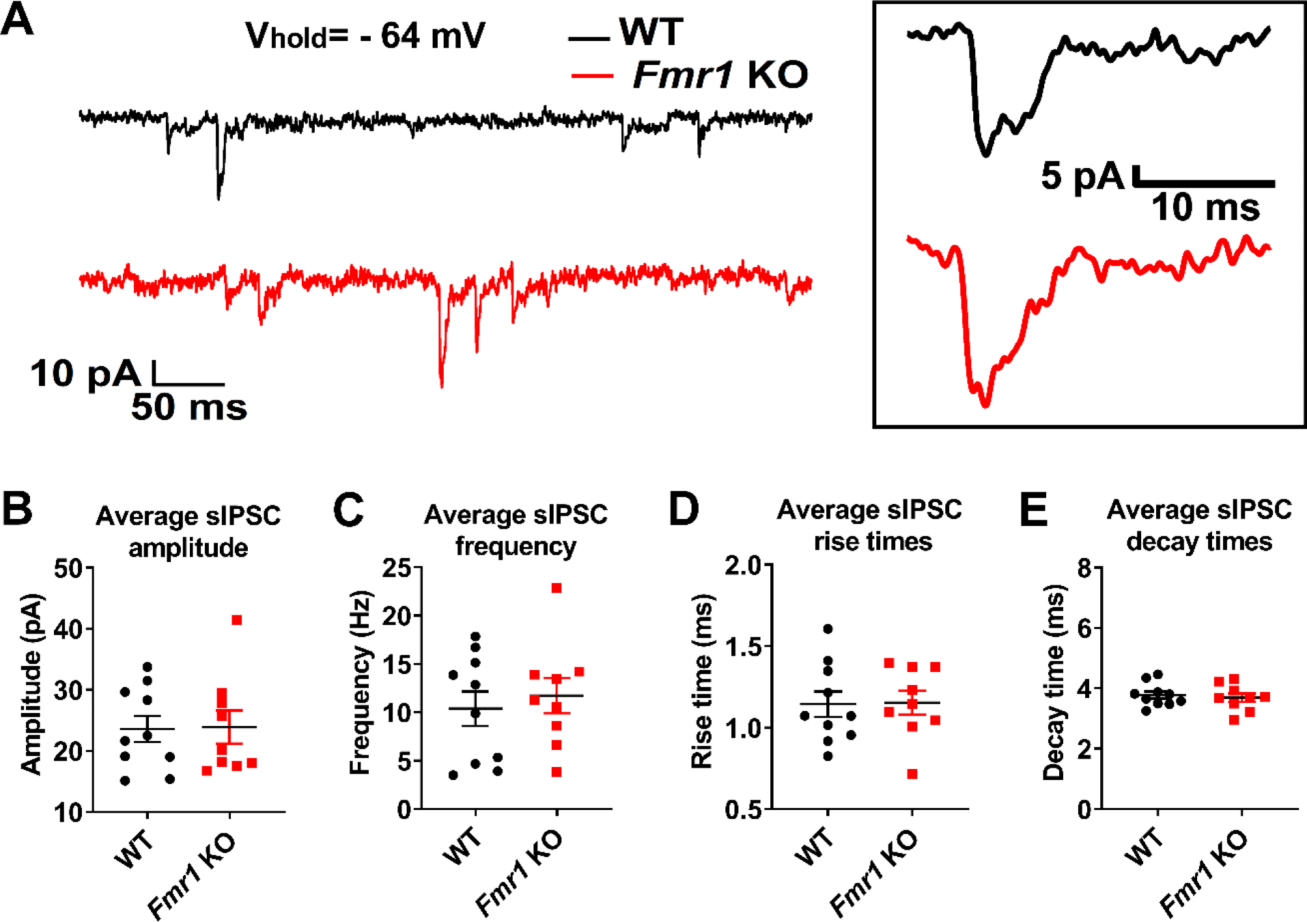
Spontaneous inhibitory postsynaptic currents (sIPSC) recordings from *Fmr1* KO mice display similar sIPSC average amplitude, frequency, and kinetics. **(A)** Representative sample traces of sIPCS recorded from *Fmr1* KO (red) and WT (black) neurons. Inset shows representative sIPSC events from *Fmr1* KO and WT cells. Graphs **(B)**, **(C)**, **(D)** and **(E)** show the per-cell average of sIPSC amplitude, frequency, rise time and decay time, respectively, for *Fmr1* KO and WT cells

At the network level, hyperexcitability alterations can depend on excitatory-inhibitory imbalance of the neuronal circuits in FXS, as has been reported in other neurodevelopmental disorders [35, 36]. Thus, using whole-cell voltage-clamp we explored excitatory and inhibitory neurotransmission in the PC of *Fmr1* KO mice by recording both excitatory (Fig. 6) and inhibitory (Fig. 8) spontaneous synaptic currents (sEPSC and sIPSC, respectively). We found no differences for sEPSC per cell in mean amplitude (Fig. 6A, B; each point corresponds to a cell; WT: 10.5 ± 1.8 pA, *n* = 9; *Fmr1* KO: 11.1 ± 1.0 pA, *n* = 10; t-test, *p* = 0.37) or frequency (Fig. 6C; WT: 3.8 ± 3.5 Hz, *n* = 9; *Fmr1* KO: 3.8 ± 4.1 Hz, *n* = 10; t-test, *p* = 0.99) between groups. Since changes in the distribution can be hidden by average calculation, we proceeded to compare the distribution of amplitudes and inter-event intervals between WT and *Fmr1* KO. We recorded a total of 2843 and 4318 events from neurons from WT and *Fmr1* KO animals, respectively, and calculated the cumulative probability distributions (CPDs) using a bootstrapping approach to address potential biases caused by imbalances in sample sizes per cell (see Methods). We observed that *Fmr1* KO neurons displayed sEPSC amplitudes distributed toward higher values (Fig. 7A; permutation-based KS (pb-KS) test *D* = 0.09 ± 0.01; *p* < 0.0001) when compared to WT controls. The CPDs for the inter-event intervals were also different between groups, as *Fmr1* KO sEPSC distribute with more probability towards shorter times (Fig. 7B; pb-KS test *D* = 0.05 ± 0.01; *p* < 0.01).

The kinetics of synaptic currents recorded at the cell body depend on how far from the soma the synaptic contacts and neurotransmitter receptors are located [37, 38], the subunit composition of the neurotransmitter receptors [39, 40], as well as other factors such as receptor clustering and transmitter clearing from the synaptic cleft [41]. Since FMRP is known to affect the expression of AMPA and GABAA receptors [42, 43] it is possible that the observed differences in the CPDs of sEPSC might arise due to differences in the types of neurotransmitter receptors expressed in these cells. Therefore, we evaluated sEPSC kinetics in WT and *Fmr1* KO cells, finding no differences for sEPSC decay times (Fig. 6C; WT: 3.7 ± 0.6 ms, *n* = 9; *Fmr1* KO: 3.3 ± 0.7, *n* = 10; t-test, *p* = 0.21) and rise times (Fig. 6D; WT: 1.5 ± 0.2 ms, *n* = 9; *Fmr1* KO: 1.3 ± 0.3 ms, *n* = 10; t-test, *p* = 0.077) between groups. However, CPD of rise and decay times of sEPSC strongly differ between WT and *Fmr1* KO, showing that rise times (Fig. 7D; pb-KS test *D* = 0.14 ± 0.01; *p* < 0.0001) as well as decay times (Fig. 7C; pb-KS test *D* = 0.10 ± 0.01; *p* < 0.0001) distribute towards faster kinetics in *Fmr1* KO cells.

**Fig. 9:**
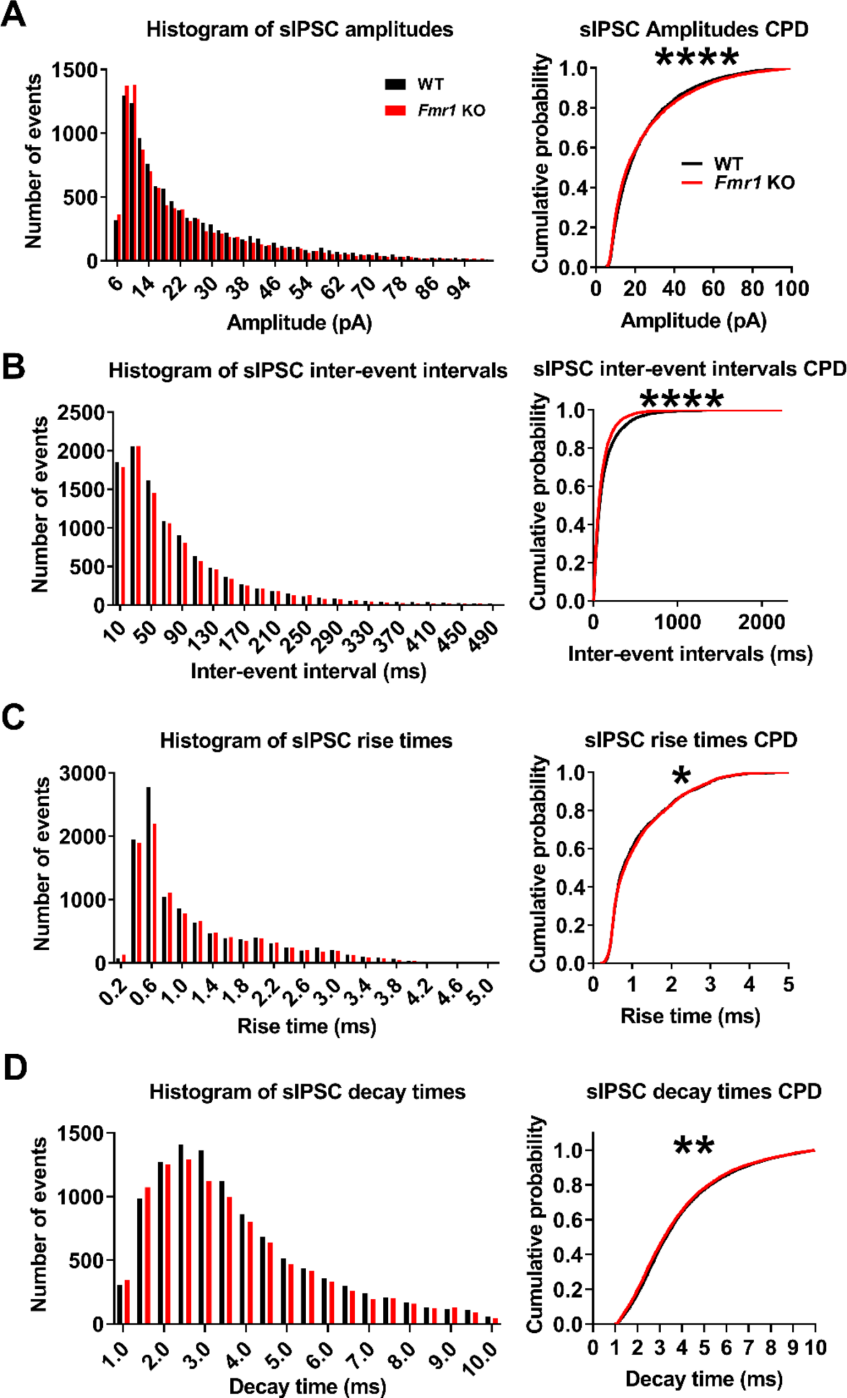
Distribution of sIPSC population´s amplitude, inter-event interval and kinetic parameters from *Fmr1* KO and WT neurons are different **(A)** Left: Histogram of sIPSC amplitudes from all WT and all *Fmr1* KO cells. Right: bootstrapped CPD graph of sIPSC amplitudes (bin = 5 pA). Graphs **(B)**, **(C)** and **(D)** show the same as in **(A)** but for inter-event intervals (bin = 20 ms), rise times (bin = 0.2 ms) and decay times (bin = 0.5 ms) respectively. (*: *p* < 0.05, **p < 0.001; ****: *p* < 0.0001)

With respect to sIPSC, there were no differences for sIPSC mean amplitude (Fig. 8B; WT: 23.6 ± 6.7 pA, *n* = 10; *Fmr1* KO: 23.9 ± 8.1 pA, *n* = 9; t-test, *p* = 0.93) and frequency (Fig. 8C; WT: 10.4 ± 5.6 Hz, *n* = 10; *Fmr1* KO: 11.7 ± 5.4 Hz, *n* = 9; t-test, *p* = 0.61) between *Fmr1* KO and WT controls. We recorded a total of 10,648 and 9949 events in WT and *Fmr1* KO neurons, respectively. As for sEPSC, we calculated the CPDs using a bootstrapping approach to evaluate possible differences in the distribution of sIPSC properties. However, CPDs of *Fmr1* KO sIPSC amplitudes were slightly shifted toward lower values (Fig. 9A; pb-KS test *D* = 0.043 ± 0.0063; *p* < 0.0001) when compared to WT controls. The CPDs for the inter-event intervals distribute with more probability towards shorter intervals (Fig. 9B; pb-KS test *D* = 0.066 ± 0.0049; *p* < 0.0001) compared to WT. There are also no differences in sIPSC decay times (Fig. 8E; WT: 3.8 ± 0.4, *n* = 10; *Fmr1* KO: 3.7 ± 0.4, *n* = 9; t-test, *p* = 0.63) nor rise times (Fig. 8D; WT: 1.1 ± 0.2, *n* = 10; *Fmr1* KO: 1.2 ± 0.2, *n* = 9; t-test, *p* = 0.93) between groups. However, CPD of sIPSC rise times distributes towards slower kinetics (Fig. 9C; pb-KS test *D* = 0.03 ± 0.01; *p* < 0.05), while the decay times (Fig. 9D; pb-KS test *D* = 0.03 ± 0.01; *p* < 0.01) CPDs distributed towards faster kinetics in *Fmr1* KO. Thus, our data suggests that there is a change in the synaptic population responsible to sustain excitatory-inhibitory balance that may favor a higher excitability in the *Fmr1* KO PC.

## Discussion

### Olfactory-guided behavior in the *Fmr1* KO mouse

Our results show that, compared to the WT group, *Fmr1* KO mice showed similar learning capabilities in simple olfactory discrimination tasks. However, these animals displayed deficiencies in processes involving higher cognitive functions, such as the ability to recall the same task 5 weeks later, and the ability to accomplish finer discrimination tasks involving complex mixtures of odorants.

There are only two prior studies that examined olfactory-cue mediated training using the *Fmr1* KO mice. Both showed that *Fmr1* KO mice can learn to discriminate between two non-structurally-related monomolecular odorants in a two-alternative force choice [12] and in a go-no go task using a similar olfactometer than the one we used in our research [11]. However, natural olfactory stimuli are constituted by complex odorant mixtures and each sniff introduces multiple airborne chemicals at the same time. Therefore, animal brains are required to separate neuronal activity patterns in order to discriminate closely related stimuli.

The question of how complex mixtures are coded and discriminated is difficult to answer and still a matter of debate. The difficulty arises from the overlapping sensory representations of olfactory neurons. Indeed, sensory neurons respond to more than one odorant and one odorant can activate more than one olfactory receptor and thus different populations of neurons [44, 45]. Therefore, for the complex mixtures found in the environment, the combinatorial activation of olfactory neurons gets more intricate. This combinatorial representation is maintained upstream in the glomerular layer of olfactory bulb [28, 46–48]. In the bulb, MC project a single apical dendrite to only one glomerulus [13, 47, 49], transmitting olfactory features coded in parallel columns of afferent activity downstream to the PC. Interestingly, the level of glomerular olfactory representations overlap is inversely correlated with the capacity of an animal to segregate olfactory targets [50]. Nonetheless, rodents can be trained to discriminate complex mixtures in a neuronal process that is believed to occur in the PC [51]. Indeed, principal neurons in the PC receive inputs from multiple MC and one MC project onto several principal neurons [52]. This convergence is thought to be required for the formation of the perceptual odor object (i.e.: the aroma of a rose) [16]. Thus, faulty afferent input to the cortex could affect how olfactory stimuli features, and ultimately olfactory perception, are coded and contribute to the olfactory deficit we observed in the *Fmr1* KO mice. Indeed, a recent work has shown that in *Fmr1* KO mice, mitral cells display enhanced basal excitation, which causes a reduction in stimulus-evoked MC responses [11]. Moreover, and in agreement with the role of FMRP regulating neuronal branching [53], MC anatomical differences have also been described in the KO, where two apical dendrites have been observed [54]. It is not clear if both dendrites terminate in one glomerulus, where it will cause an overflow of information to the PC or if they terminate onto different glomerulus which will force the system to an early integration of the odor mixture affecting, in both cases, olfactory discrimination of complex odor mixtures.

On the other hand, previous experience with an olfactory stimulus could promote pattern completion and perceptual stability. Pattern completion is a process that is largely mediated by activity integration at the PC network allowing the recognition of the stimulus even when some features are presented in an occluded or modified manner. At a neuronal level, pattern completion allows for the completion of the neural activity map encoding the identity of the stimulus, by comparing the present version of the stimulus with a memory of it. Moreover, it has been suggested that the blend we used in our experiments (10 C and 10 C-1) activates cortical ensembles that will initially overlap hindering discrimination, and that experience with both olfactory stimuli will promote neuronal activity pattern separation later in the learning process [16, 24, 55]. The PC is an allocortex organized in three layers. Layer Ia is where the axonal projection of mitral/tufted cells synapse onto the dendritic arbors of layer II and III pyramidal neurons [33]. A distinct feature of the PC is the intrinsic intracortical association fibers and the strong long-term potentiation (LTP) they exhibit [56, 57]. As groups of principal neurons are simultaneously activated by the combinatorial pattern of bulbar inputs, an ensemble of co-active neurons can become a long-lasting memory engram by the strengthening of intrinsic association fibers. Re-exposure to an incomplete version of the original complex stimulus, could then drive the activation of the whole engram [16, 24, 55] and to pattern completion. Importantly, a fine-tuned equilibrium of LTP may be required to allow for pattern completion, but also for pattern separation. Massive LTP could impair olfactory discrimination and pattern separation. In *Fmr1* KO mouse LTP appears to be no differences compared to control mice in the PC and hippocampus [58] in adult mice, however, this seems to depend on the strength of the stimulation protocol [59]. In this complex scenario, differences in neuronal excitability could be a factor contributing to pattern completion or olfactory memory deficits observed in *Fmr1* KO.

### The hyperexcitable phenotype of layer II principal cells in the *Fmr1* KO mice piriform cortex

Our results demonstrate that principal cells in layer II of *Fmr1* KO mice PC have a hyperexcitable phenotype that is not accompanied by changes in their passive membrane properties, but it is mainly sustained by anomalies in voltage-dependent processes. Indeed, analysis of active membrane properties revealed differences in AP threshold and AP overshoot. A lower AP threshold in *Fmr1* KO cells is consistent with the hyperexcitable phenotype, meaning that cells will engage in firing behavior after smaller depolarization compared to control neurons. This result is in line with previous research that found lower AP threshold in the medial prefrontal cortex layer 5 pyramidal neurons of *Fmr1* KO mice [60]. While we did not address in detail the physiological mechanisms underlying altered AP threshold and F-I relationship, we will discuss some alternatives. For instance, the hyperpolarized threshold could depend on differences in subthreshold voltage-dependent current, as a reduced conductance of somatic slowly-inactivating (D-type) K^+^ channels [61]. However, changes in this conductance would be expected to alter the latency of the first AP, which is not the case here. On the other hand, AP threshold could be modified by alterations in persistent Na^+^ current as demonstrated in *Fmr1* KO entorhinal cortex principal cells [62].

On the other hand, the diminished AP overshoot may be related to changes in fast-activating K^+^ or Ca^2+^ currents or alterations in the activation/inactivation of Na^+^ channels. Voltage-activated K^+^ channels Kv3 could affect overshoot and explain the trend to a higher fAHP, however, this is most probably not related to the increase in frequency, as Kv3 fast deactivation kinetics is expected to trigger firing at much higher frequencies (more than 100 Hz) [29]. fAHP can also be mediated by BK channels [63], which are expressed in PC neurons [64] and are directly modulated by FMRP [65]. Additional experiments will contribute to clarifying the molecular components mediating the observed changes. Finally, it is worth mentioning that a lower GABAergic tone has been reported in the amygdala and subiculum of the *Fmr1* KO compared to WT animals [66, 67] that could impact the cell input resistance. Therefore, future studies using pharmacology could take this factor into account to dissect the role GABAergic tone plays in the hyperexcitability phenotype we observe.

### Excitatory-inhibitory balance in PC layer II neurons of *Fmr1* KO mice

In this study we found that, on average, neither spontaneous excitatory nor inhibitory synaptic activity show alterations in amplitude, frequency, or kinetics between groups. Interestingly, when the distribution of these properties was analyzed, we found changes in amplitude and inter-event intervals in subpopulations of events. The amplitude distribution of sEPSC distributed toward larger amplitudes and inter-event intervals was slightly shifted toward shorter times in *Fmr1* KO mice. As spontaneous events comprise miniature postsynaptic currents (mPSC) occurring by stochastic release in the contents of a single vesicle as well as simultaneous AP-mediated release of single or multiple vesicles, the shifts in CPD could be related in part to the increased intrinsic excitability of the same layer II neuron population, affecting associative synaptic transmission. Therefore, the shift in amplitude distribution to the right could be mediated by both an increase in the released quantum content as well as by a higher number of AMPA receptors mediating mEPSC on principal layer II PC neurons [68].

On the other hand, although there are no differences for average rise and decay time of sEPSC between WT and *Fmr1* KO, they were distributed with higher probability towards shorter decay and rise times. Many changes in the dendritic properties of *Fmr1* KO cells, such as the type, number, clustering of receptors, dendrite architecture, distance from the soma, and conductance could explain the altered kinetic properties observed in our experiments [39, 41]. Moreover, since FMRP has been shown to have such a pleiotropic impact in these properties, all these possibilities are much in play to explain these alterations. More specifically, sEPCS decay and rise time shifting toward faster kinetics might be due to the enrichment of activity of synapses located more proximal to the soma, since events occurring more distant from the soma undergo greater filtering, appearing with slower kinetics than proximal ones [38]. Furthermore, inputs from association fibers are closer to the soma compared with afferent inputs from the LOT that more commonly arrives in layer Ia [15], supporting the hypothesis that the changes in the distribution for sEPSC depend on altered excitability of inputs coming from associative fibers rather than from LOT connections [33]. Also, association presynaptic terminals are sensitive to GABAB receptor modulation of glutamate release, in contrast to afferent input from the LOT, and there is evidence of a loss of GABAB receptor activity in other brain areas in *Fmr1* KO mice [69]. This could mediate an increase in release probability from association fibers, contributing to our observed changes in sEPSC properties. Finally, AMPA receptors lacking the GluA2 subunit mediate sEPSC with faster decay kinetics and increased amplitude currents [41]. Simultaneously, evidence from *Fmr1* KO mice model and FXS-derived pluripotent human cells indicate altered expression of GluA2-containing AMPA receptors [70, 71]. These findings suggest that the altered sEPSC properties distribution observed in our data might be determined by changes in GluA2-lacking AMPA receptors expression patterns. Interestingly, these Ca^2+^-permeable receptors are critically involved in both homeostatic and Hebbian synaptic plasticity [72, 73] and thus, it would be worthwhile to explore this possibility further. Overall, the observed alterations in the amplitude and kinetics of excitatory inputs may impact on spatiotemporal dendritic integration and synaptic plasticity in the PC.

Regarding sIPSC analysis, we also found no differences in the average response when *Fmr1* KO neurons were compared to WT neurons. When the events distributions was analyzed, we found a shift to the left in inter-event intervals, as was the case for sEPSC. As we did not study interneuron intrinsic excitability, this result is even more difficult to interpret, as it may in principle depend on both synaptic and intrinsic properties. In general, there is agreement regarding a dampening of phasic and tonic inhibitory drive in the *Fmr1* KO mouse, leading to a hyperexcitable phenotype [74, 75]. The possibility that in the PC the inhibitory network might be hyperexcited, opens new questions worth pursuing. Furthermore, comparison of amplitude distributions suggests that different groups of events might be modified slightly and in opposite ways. Which regards to kinetics parameters, the changes were mild and may not have biological significance. Anyhow, it is worth mentioning that there might be changes in the proportion of GABA_A_ receptor subunits [39] in the PC of the *Fmr1* KO. In fact, evidence of reduced expression of GABA_A_ receptor subunits in the cortex of *Fmr1* KO mice have been widely reported [76], as well as direct FMRP interaction with delta GABA_A_ receptor subunit. Thus, further research looking at these properties in detail would be necessary to elucidate the origin and significance of the changes in sIPSC distributions.

Altogether, our results show that there are changes in sEPSC and sIPSC properties distribution in the *Fmr1* KO that could partly underlie the observed synaptic hyperexcitability phenotype of the PC of *Fmr1* KO mice. Nonetheless, more research needs to be done to determine the mechanisms and biological relevance of these changes to the systemic and behavioral phenotype.

## Data Availability

The datasets generated during the current study are available from the corresponding author on reasonable request.
